# Improving prognostic performance in resectable pancreatic ductal adenocarcinoma using radiomics and deep learning features fusion in CT images

**DOI:** 10.1038/s41598-021-80998-y

**Published:** 2021-01-14

**Authors:** Yucheng Zhang, Edrise M. Lobo-Mueller, Paul Karanicolas, Steven Gallinger, Masoom A. Haider, Farzad Khalvati

**Affiliations:** 1 Department of Medical Imaging, University of Toronto, 686 Bay Street, Toronto, ON M5G 0A4 Canada; 2 Department of Diagnostic Imaging and Department of Oncology, Faculty of Medicine and Dentistry, Cross Cancer Institute, University of Alberta, Edmonton, AB Canada; 3 Department of Surgery, Sunnybrook Health Sciences Centre, Toronto, ON Canada; 4 Lunenfeld-Tanenbaum Research Institute, Sinai Health System, Toronto, ON Canada; 5 Joint Department of Medical Imaging, University Health Network, University of Toronto, Toronto, ON Canada; 6 Department of Diagnostic Imaging and Research Institute, The Hospital for Sick Children, Toronto, ON Canada; 7 Department of Mechanical and Industrial Engineering, University of Toronto, Toronto, ON Canada

**Keywords:** Cancer imaging, Paediatric cancer, Tumour biomarkers, Biomarkers, Biomedical engineering

## Abstract

As an analytic pipeline for quantitative imaging feature extraction and analysis, radiomics has grown rapidly in the past decade. On the other hand, recent advances in deep learning and transfer learning have shown significant potential in the quantitative medical imaging field, raising the research question of whether deep transfer learning features have predictive information in addition to radiomics features. In this study, using CT images from Pancreatic Ductal Adenocarcinoma (PDAC) patients recruited in two independent hospitals, we discovered most transfer learning features have weak linear relationships with radiomics features, suggesting a potential complementary relationship between these two feature sets. We also tested the prognostic performance for overall survival using four feature fusion and reduction methods for combining radiomics and transfer learning features and compared the results with our proposed risk score-based feature fusion method. It was shown that the risk score-based feature fusion method significantly improves the prognosis performance for predicting overall survival in PDAC patients compared to other traditional feature reduction methods used in previous radiomics studies (40% increase in area under ROC curve (AUC) yielding AUC of 0.84).

## Introduction

In the past decade, as an emerging field, radiomics has been developed to extract more information from medical images for improved diagnosis and prognosis of cancer. As a quantitative approach, radiomics comprises of the extraction and analysis of quantitative medical imaging features and establishing correlations between these features and clinical outcomes such as patient survival^1–5^. Several radiomic features have been found to be significantly associated with various clinical outcomes in multiple cancer sites such as lung, pancreas, and kidney^2,6–12^.

In the past few years, the pipeline for traditional radiomics analysis has been established^1,2,9,13^. This traditional pipeline consists of four steps: image acquisition, region of interest (ROI) segmentation or annotation, feature extraction, and building a predictive model. As the core of this pipeline, radiomics features are extracted from medical images using predefined mathematical equations^14^. These engineered equations have been designed to capture different characteristics of images^15^. For example, first-order features measure the distribution of pixel intensities while second-order features are based on matrices including grey-level co-occurrence matrix (GLCM) and grey-level run length matrix (GLRLM) and extract texture information^14^. Efforts have been made to standardize the feature banks by implementing open source libraries such as PyRadiomics^15^. In these feature banks, thousands of engineered features from different classes can be extracted from 2D or 3D medical images^15^. These features can be further tested for their associations with clinical outcomes such as overall survival, recurrence, or genetic mutations^4,8,16,17^. Several cross-cohort and multi-centre studies have also shown that several PyRadiomics features are robust to different scanners and clinician annotations^8,15,18,19^.

Despite recent progress, the traditional radiomics analytics pipeline has a few drawbacks. First, the equations of features are predefined, and many formulas are similar. Thus, some radiomics features are highly correlated with each other. As a result, if a feature was found to be significantly associated with a certain clinical outcome, other highly correlated features may be significant as well. Consequently, while the high dimension of significant features increases the complexity of the prognostic model, there is no corresponding increase in performance. Second, testing radiomics features one by one increases the family-wise error rate (FWER), which is the probability of making one or more false discoveries. Previous publications have pointed out that several radiomics studies lacked multiple testing control and hence, some discovered significant features may be the result of type I errors^20,21^. These shortcomings in the traditional radiomics analytics pipeline have inspired new research which takes advantage of the recent progress in deep learning and convolutional neural networks (CNNs) to improve the performance of the predictive models.

CNNs are one of the most frequently used deep learning architectures in computer vision^22^. CNNs apply a series of convolution operations on input images, preserving the spatial relationship between pixels and mapping these relationships onto outputs. During the training phase, parameters of the convolution operations are tuned based on the outcome. Consequently, convolution layers can capture information specifically related to the classification task (e.g., outcome prediction) at hand. In medical imaging, this allows generating customized feature maps for specific modalities or diseases, which further improves performance^23,24^. However, training CNN parameters requires a large sample size, which is usually not available in typical medical imaging research settings. To overcome this limitation, transfer learning-based feature extraction has been proposed^25–27^.

Transfer learning was developed based on an assumption that the structures of CNNs are similar to the mechanism of the human visual cortex^22,28^. The top layers of CNNs can extract general features from images, while the deeper layers are more specific to the target^22^. Pretraining CNNs using large image datasets such as ImageNet helps the model to learn how to extract general features^29,30^. Since many image recognition tasks are similar, the top layers of the network can be transferred to another target domain^26^. On the other hand, deeper layers of CNNs can extract “higher-order” information which is associated with the target outcome. Thus, if the target domain is similar to the pretrained domain, deeper layers can also be transferred to extract features^25,31^.

Deep learning and transfer learning-based feature extraction have shown promising results in cancer assessment^31–33^. Furthermore, it has also been shown that combining predefined features with deep learning-based features can improve the performance in the prognosis of Glioblastoma Multiforme^31^. To gain a deeper understanding of the relationship between traditional radiomics and transfer learning features, it is crucial to map the correlation between these two sets of features. In addition, it is imperative to develop an optimal feature fusion pipeline that can exploit the prognostic information from both feature sets to improve the overall performance of the model.

The aim of this study was to assess the complementary prognostic information of predefined radiomic features and transfer learning features for overall survival in CT scans of Pancreatic Ductal Adenocarcinoma (PDAC) patients. Using CT images from PDAC patients, we mapped the association between PyRadiomics and a set of transfer learning features and showed the correlation among the two classes of features. Next, we applied four existing feature fusion and reduction methods, which include principal component analysis (PCA), Boruta^34^, feature-wise selection using the Cox Proportional Hazards Model (CPH)^35^, and LASSO^36^, to combine the predefined radiomic features with transfer learning features for the prognosis of overall survival in PDAC patients. We then proposed a novel pipeline for combining predefined radiomics features and transfer learning features using a risk-score based model and compared its performance to aforementioned four existing feature fusion and reduction methods in an independent test cohort.

## Methods

### Dataset

Two cohorts from two independent hospitals consisting of 68 (training cohort) and 30 patients (test cohort) who had pre-operative contrast-enhanced CT available for analysis were enrolled in this retrospective study. All patients underwent curative-intent surgical resection for PDAC from 2008–2013 to 2007–2012 for both cohorts, respectively, and they did not receive other neo-adjuvant treatment. CT scans were performed on Toshiba, Aquilion (training cohort) and GE Medical Systems, LightSpeed VCT (test cohort) scanners using 2–3 mm slice thickness in the portal venous phase without advanced dose reduction algorithms.

Survival data were collected retrospectively (training cohort: 52 death vs. 16 survival, test cohort: 15 death vs. 15 survival at the end of follow-up). The median follow-up date was 21 months (range: 101 days to 1890 days) and 19 months (range: 109 days to 2569 days) for the training and test cohorts, respectively. We selected the two-year survival as the primary outcome, which was determined by the last follow-up date or date of death 2 years after surgery (Training cohort: 38 death vs. 30 survival, test cohort: 11 death vs. 19 survival). Further demographic information about these two cohorts can be found in Table 1^8^. To exclude the effect of postoperative complications on the prognosis, the patients who died within 90 days after surgery were excluded. An in-house developed region of interest (ROI) contouring tool (ProCanVAS)^37^ was used by an experienced radiologist to annotate ROIs. The reader contoured the ROIs blind to the outcome.

**Table 1:** Demographic information of training and test cohorts^8^.

	Training cohort	Test cohort
**Age (years)**		
Mean ± standard deviation	65 ± 11	69 ± 8
**Sex**		
Male/female/total	35/33/68	13/17/30
**Tumour size (diameter—cm)**		
Mean ± standard deviation	4.34 ± 1.47	3.76 ± 0.97
**Grade**		
G1/G2/G3/G4/total	17/44/6/1/68	3/19/8/0/30

### Ethics approval and consent to participate

For the training cohort, University Health Network Research Ethics Boards approved the retrospective study and informed consent was obtained. For the test cohort, the Sunnybrook Health Sciences Centre Research Ethics Boards approved the retrospective study and waived the requirement for informed consent. All methods were performed in accordance with the relevant guidelines and regulations of both institutions.

### Radiomics feature extraction

Pre-defined radiomic features were extracted using the PyRadiomics library (version 2.0.0) in Python^15^. To ensure that features were extracted from tumour regions exclusively, voxels with Hounsfield unit (HU) < -10 and > 500 were excluded to eliminate fat and stents from the feature values. A threshold of 500 would only exclude large parts of blood vessels in the portal venous phase which are not part of the tumor contour. These are normal structures that if included would confound analysis. This threshold, however, would not exclude tumor neovasculature or hyperenhancing subcomponents in the tumor which do not reach such a high attenuation level. In total, 1,428 radiomic features were extracted for both cohorts from the contoured ROIs. Details of the extracted features are listed in Table 2.

**Table 2:** Number of radiomics features extracted for different feature classes and image filters.

Filter/features	First-order	GLCM	GLDM	GLRLM	GLSZM	NGTDM	Shape	Total
Exponential	16	0	11	12	7	0	0	46
Gradient	18	23	14	16	16	5	0	92
lbp	56	0	44	48	28	0	0	176
Logarithm	18	23	14	16	16	5	0	92
Original	18	23	14	16	16	5	12	104
Square	18	23	14	16	16	4	0	91
Squareroot	18	23	14	16	16	5	0	92
Wavelet	144	184	112	128	128	39	0	735
Total	306	299	237	268	243	63	12	1428

*GLCM* grey level co-occurrence matrix, *GLDM* grey level difference matrix, *GLRLM* grey level run length matrix, *GLSZM* gray level size zone, *NGTDM* neighboring gray tone difference matrix.

### Transfer learning feature extraction

**Figure 1:**
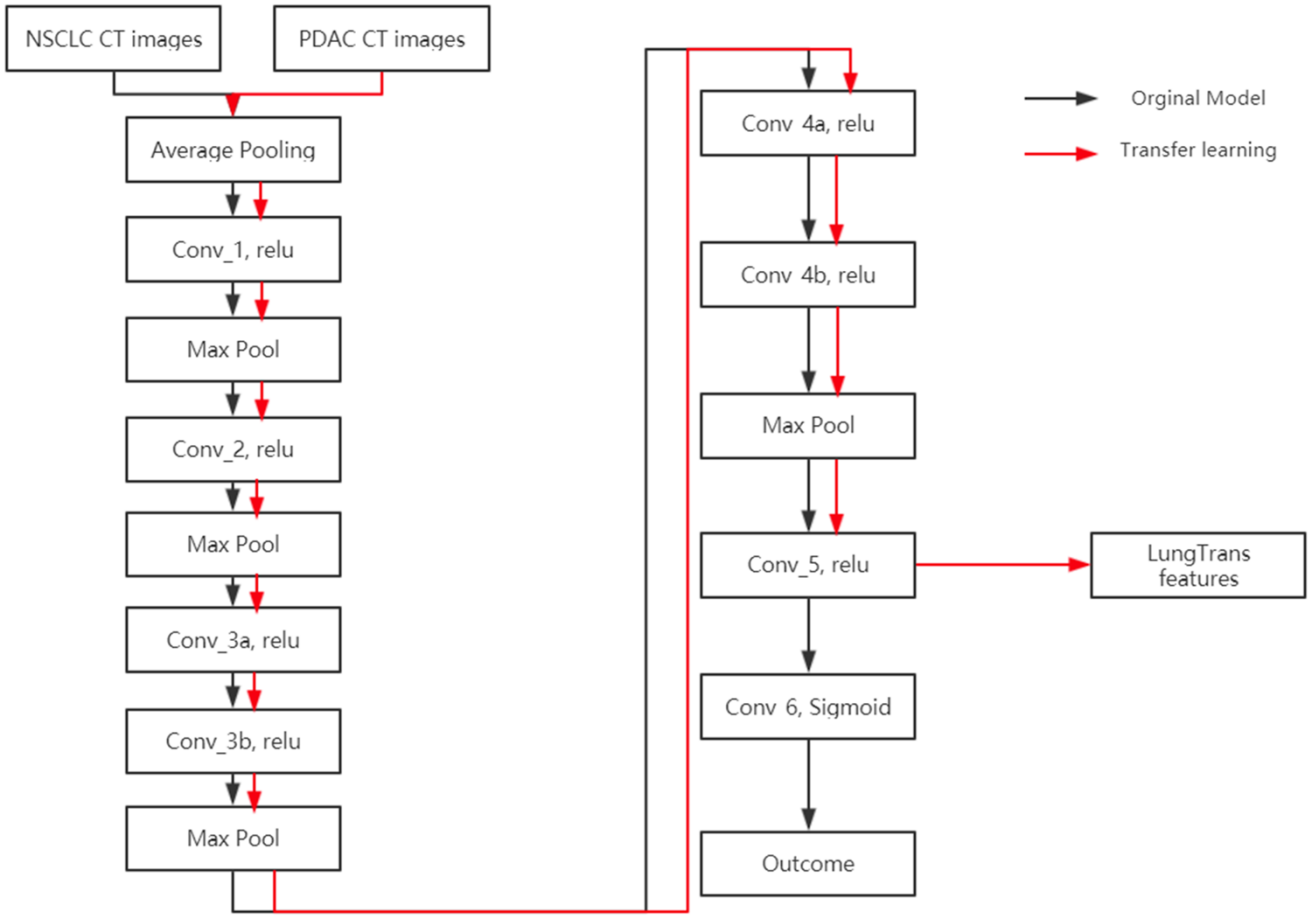
Architecture of the 8-layer CNN used to extract LungTrans Features.

Transfer learning features were extracted using a CNN model (LungTrans) pretrained by Non-Small Cell Lung Cancer (NSCLC) CT images^38^. The NSCLC dataset was published as Lung Nodule Analysis (LUNA16) challenge with CT images from 888 patients^39^. Images were extracted from the largest contoured ROI from each patient without preprocessing. All input ROIs were resized to 32 $\times \hspace{0.17em}$32 greyscale. Given that the shape of the ROI is not rectangular, the region outside of the ROI was set as black. Using this dataset, an 8-layer CNN (LungTrans) was trained de novo with batch size 16 and learning rate 0.001, with the architecture shown in Fig. 1^40^ Every convolutional layer has Kernel size of 3 $\times \hspace{0.17em}$3 with stride of 1 with zero padding except for Conv_5 layer which has 2 $\times \hspace{0.17em}$2 kernel size and stride of 1 without padding. All the Max Pooling layers have 2 $\times \hspace{0.17em}$2 kernel size.

The process of transfer learning varies depending on the similarity of the pretrained domain and target domain. If the pretrained and target domains are different (e.g., natural images vs. CT pancreatic images), features will generally be extracted from upper layers for better generalization. However, if the pretrained and target domains are similar (e.g., they share the same imaging modality, similar resolution, and similar outcome), features can be extracted from deeper layers. In this study, since the pretrained and target domains are similar (lung and pancreatic CT), features were extracted from the Conv_5 layer which is a deep layer just before classification layers. Feeding the LungTrans CNN with contoured PDAC CT images with the same settings as the pretrained domain (32 $\times \hspace{0.17em}$32 greyscale ROI images with black background), 64 LungTrans features were extracted. After eliminating 29 LungTrans features with zero variance, 35 LungTrans remaining features were used in this study.

### Correlation

To investigate the correlation between the features extracted using traditional radiomics pipeline (PyRadiomics) and transfer learning (LungTrans), Pearson correlation coefficients were calculated for each pair of feature sets in the training cohort (n = 68). The mean absolute correlation coefficient was calculated for each feature set (PyRadiomics and LungTrans). The distributions of the correlation coefficients were also calculated.

### Proposed prognosis model

**Figure 2:**
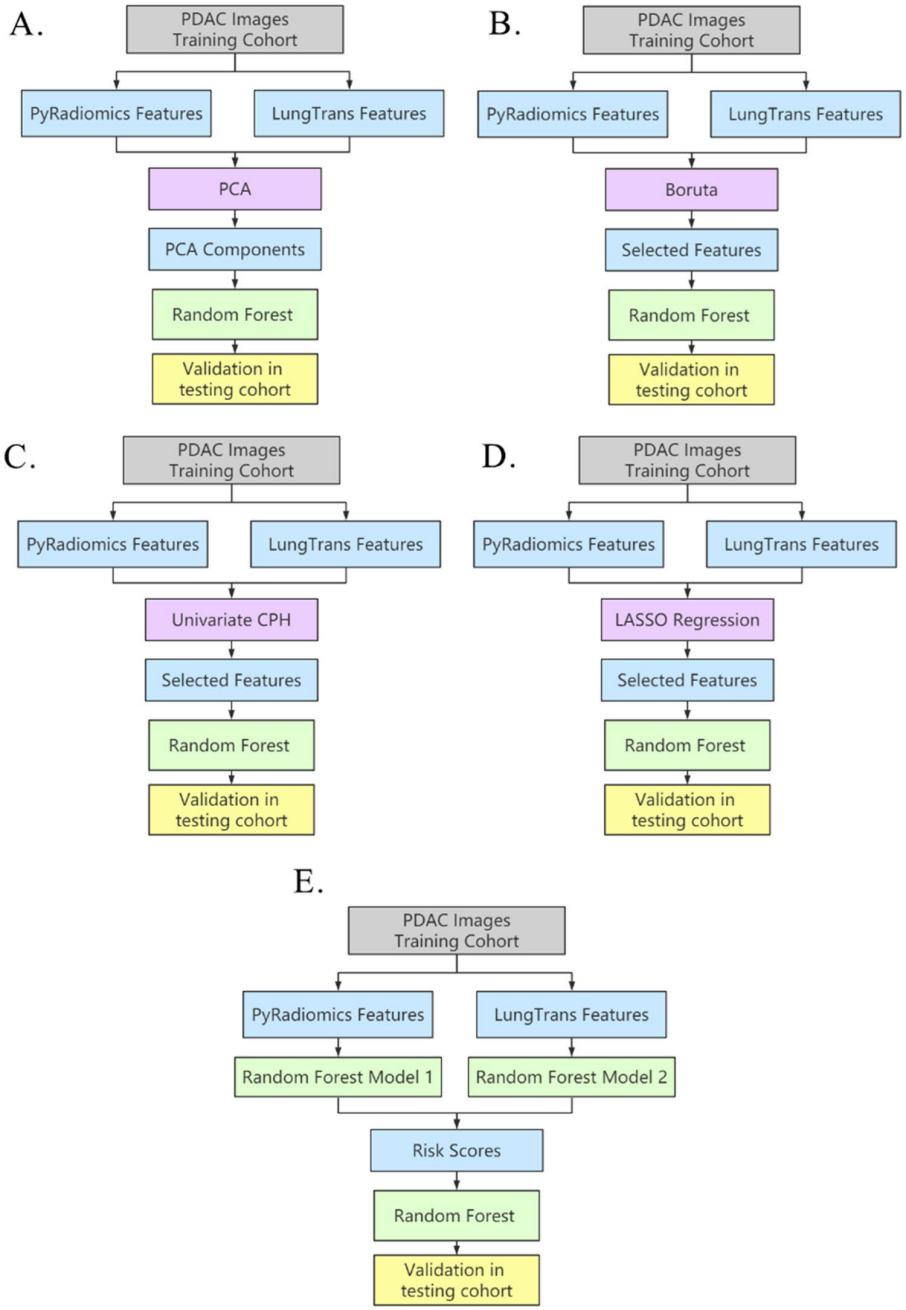
Pipelines for different feature reduction/fusion methods. (**A**) Unsupervised feature fusion using PCA. (**B**) Supervised feature reduction using Boruta. (**C**) Supervised feature reduction using Cox-Regression. (**D**) Supervised feature reduction using LASSO Regression. (**E**) The proposed risk-score based feature fusion method.

To investigate the optimal feature reduction and fusion methods, we first trained four prognosis models using CT images from the training cohort (n = 68) and validated them in the test cohort (n = 30) targeting a two-year survival. In each model, features from Pyradiomics and LungTrans were fused or selected in the training cohort using PCA, Boruta^34^, feature-wise reduction through CPH^35^, or LASSO^36^ method. These selected/fused features were then used to train Random Forest-based prognosis models (number of trees to grow (ntree) = 500, number of randomly sampled variables as candidates at each split (mtry) varies depending on the setting that had the best performance in the training cohort). These prognosis models were further validated in the test cohort. The pipelines of four traditional feature fusion/reduction algorithms including PCA, Boruta^34^, CPH-based feature reduction^35^, and LASSO^36^ are shown in Figs. 2A–D, respectively. In the following, each method is described in detail.

A. Unsupervised feature fusion using PCA: Features from two feature banks were fused using PCA, generating 30 components. Next, these components were used to build a model (Random Forest, mtry = 2) in the training cohort, which was then evaluated in the test cohort.
B. Supervised feature reduction using Boruta. Boruta identified prognostic features which were then used to build a prognosis model (Random Forest, mtry = 2) in the training cohort. The model’s performance was validated in the test cohort.
C. Supervised feature reduction using Cox-Regression. Each feature was tested using univariate Cox-regression in the training cohort. Significant features were then used to build a prognosis model (Random Forest, mtry = 310), which was validated in the test cohort.
D. Supervised feature selection using Correlation cut-off and LASSO Regression. In the training cohort, features with correlation coefficients higher than 0.7 were removed. The remaining features were reduced using LASSO logistic regression with optimized lambda. The features with nonzero coefficients in LASSO regression in the training cohort were selected to build the Random Forest model (mtry = 2), which was then evaluated in the test cohort.

Our proposed risk score-based method is illustrated in Fig. 2E. First, using the training cohort, two different Random Forest classification models were trained separately using each of the two feature banks (PyRadiomics and LungTrans) through tenfold cross validation^41^. Each of these models was then used to produce the probability of death for every patient in the training cohort through tenfold cross-validation. At this point, each patient in the training cohort would have two probabilities (training risk scores) of death based on the two feature banks (PyRadiomics and LungTrans). Similarly, feeding these two random forest models (trained using the entire training cohort) with PyRadiomics features and LungTrans features in the test cohort, two risk scores were generated for each patient in the test cohort (test risk scores). We then used these two training risk scores to train another Random Forest-based prognosis model in the training cohort and validated the model in the test cohort using the test risk scores.

To address the imbalanced outcome in the training cohort, SMOTE algorithm^42^ was applied in the training process of all five models as it has been shown that SMOTE’s performance is comparable to that of more recent balancing methods such as ADASYN^43^. The following settings were used for SMOTE algorithm:

- k (number of nearest neighbours used to generate the new examples of the minority class) = 5.
- perc.over = 200, perc.under = 200 (a common default setting to balance the amount of over-sampling of the minority class and under-sampling of the majority class).

The area under the ROC curve (AUC) was used to measure the performance of these five approaches^44^. Youden’s J statistics were used to identify the optimal threshold for sensitivity and specificity^45^. DeLong tests were applied to test the difference between the AUCs of different models. The classification modeling, calculation of AUC, and DeLong tests were performed using the “caret”, “survival”, and “pROC” package in R (Version 3.5.1)^46–48^.

## Results

### Correlation analysis between predefined and deep radiomic features

Within each feature bank, the average absolute values of Pearson correlation coefficients of 1,428 PyRadiomics and 35 LungTrans features were 0.27 (standard deviation: 0.23) and 0.32 (standard deviation: 0.32), respectively. The average absolute correlation coefficient between PyRadiomics and LungTrans features was 0.17 (standard deviation: 0.18). The weak linear relationship between PyRadiomics and LungTrans features suggest that the LungTrans features may harbor new information that PyRadiomics doesn’t capture.

**Figure 3:**
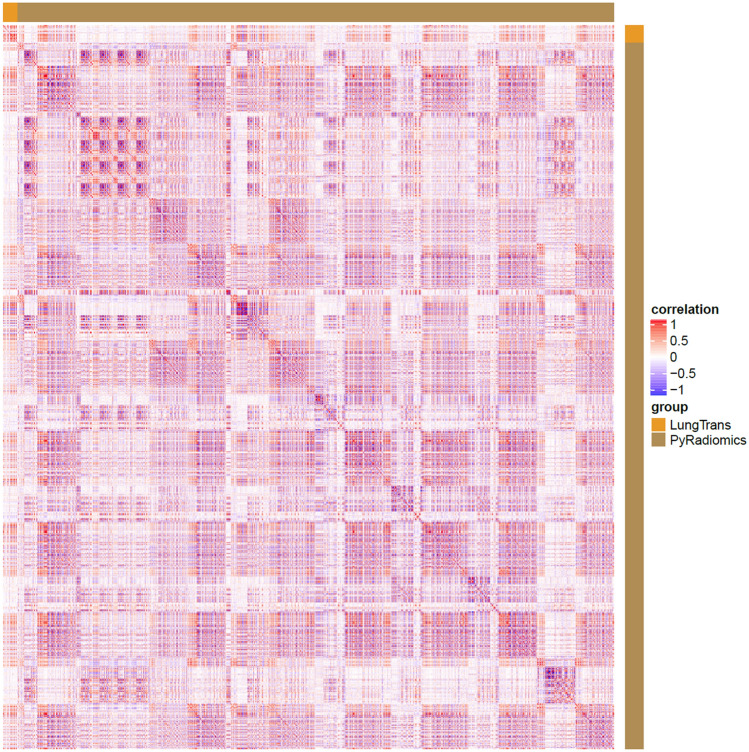
Correlation heatmap of PyRadiomics and LungTrans features.

The heatmap in Fig. 3 shows the correlation details between the two feature sets. Each dot in Fig. 3 represents a correlation coefficient. White colour indicates that the coefficient is 0, while red and blue dots represent positive or negative correlations. There are several colour blocks in PyRadiomics vs. the PyRadiomics region, indicating high correlations among the PyRadiomics features. Several colour bands in the PyRadiomics vs. LungTrans region also suggest that some LungTrans features may have strong linear relationships with PyRadiomics features.

**Figure 4:**
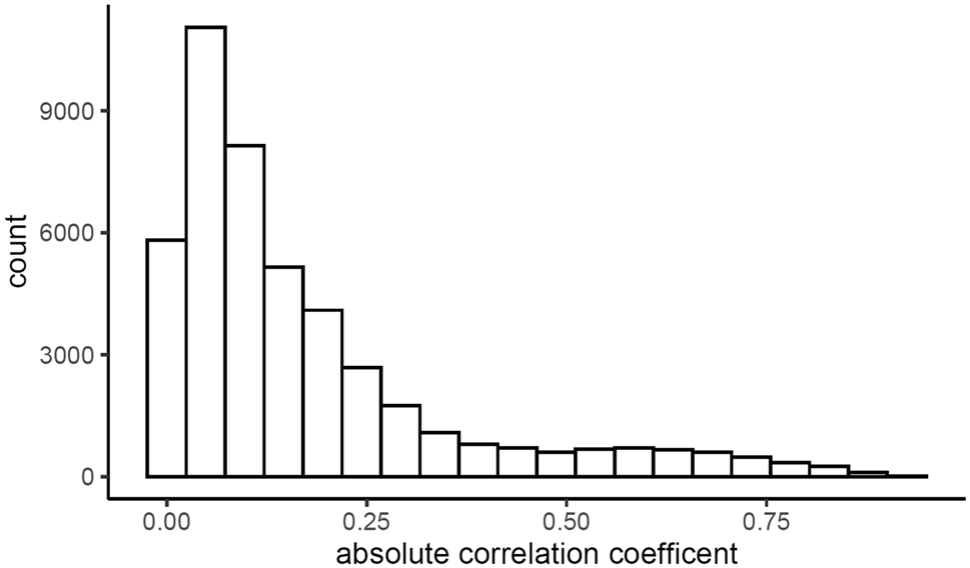
Histogram of absolute correlation coefficients between PyRadiomics and LungTrans.

The distribution of the correlation coefficients (in absolute value) is displayed in histogram form in Fig. 4. As illustrated by a skewed distribution, most of the predefined and deep radiomic features have weak correlations with one another. However, strong linear associations exist between certain features given the high correlation coefficients (> 0.70)^49^. More details for the correlation between PyRadiomics and Transfer Learning features can be found in Table 3, where the average absolute values of correlation coefficients were calculated for each type of filter and feature.

**Table 3:** Mean absolute correlation coefficients between PyRadiomics and LungTrans features across different types of filters and features.

Filter/features	First order	glcm	gldm	glrlm	glszm	ngtdm	Shape
Exponential	0.18		0.06	0.08	0.07		
Gradient	0.32	0.32	0.22	0.20	0.27	0.31	
lbp	0.08		0.06	0.08	0.07		
Logarithm	0.14	0.13	0.11	0.12	0.10	0.11	
Original	0.24	0.23	0.16	0.17	0.19	0.27	0.06
Square	0.27	0.42	0.24	0.25	0.32	0.36	
Square root	0.19	0.18	0.14	0.15	0.14	0.19	
Wavelet	0.26	0.18	0.14	0.14	0.14	0.16	

### Performance of the proposed prognosis model

The performances of four existing feature reduction methods (PCA, Boruta, feature-wise selection through CPH, and LASSO) were compared to that of the proposed risk score-based prognosis model. PCA method generated 30 components in the training cohort that represent the 95% variance in the original 1463 features from the PyRadiomics (1428 features) and LungTrans feature banks (35 features). In 100 iterations, Boruta feature reduction method selected only 1 feature in the training cohort, which was from PyRadiomics feature bank (Wavelet GLDM Small Dependence Low Gray Level Emphasis), with a cut-off at 0.05 (*p*-value cut-off for the Boruta method). CPH method identified 310 features associated with overall survival in the training cohort. Particularly, as shown in Table 4, 308 of them belong to the PyRadiomics feature bank, while LungTrans contributed with only 2 features. While some of the PyRadiomics features have been previously identified for PDAC prognosis (e.g., SumEntropy^8^), other well-known features such as ROI size was not significant. In the LASSO model, 14 features were identified as the potential prognostic biomarkers (3 features from LungTrans, and 11 features from PyRadiomics). Our proposed risk score-based model utilized the probabilities of the two individually trained Random Forest models. The performance of these five models was measured using the area under the ROC curve (AUC) for overall survival in the test cohort.

**Table 4:** Significant PyRadiomics features in univariate CPH across different types of filters and features.

Filter/feature	First order	glcm	gldm	glrlm	glszm	ngtdm	Shape	Total
Exponential	0	0	0	1	0	0	0	1
Gradient	4	11	5	7	6	1	0	34
Local binary pattern	9	0	0	4	0	0	0	13
Logarithm	1	0	1	0	2	1	0	5
Original	6	12	5	7	7	1	1	39
Square root	5	11	4	2	6	2	0	30
Wavelet	50	67	15	30	19	5	0	186
Total	75	101	30	51	40	10	1	308

In the validation (test cohort), the AUCs for PCA, Boruta, CPH, and LASSO methods were 0.60 (95% Confidence Interval (CI): 0.37–0.82), 0.60 (95% CI: 0.38–0.81), 0.55 (95% CI: 0.32–0.77), and 0.50 (95% CI: 0.28–0.72), respectively. The proposed risk score-based method produced the highest AUC (AUC of 0.84, 95% CI: 0.70–0.98).

**Figure 5:**
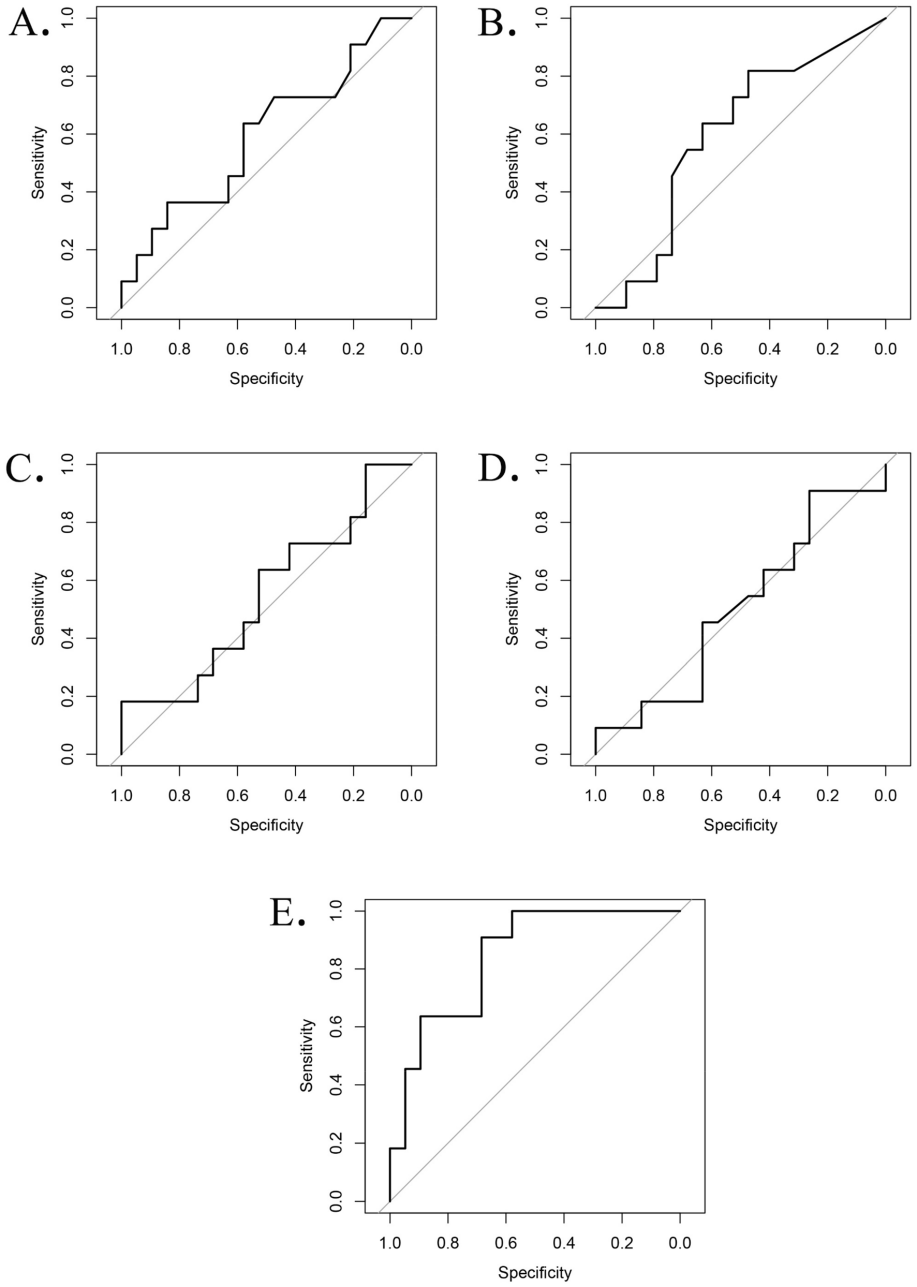
ROC curves of models using four feature reduction/fusion methods. (**A**) ROC curve for PCA based fusion method, AUC = 0.60, specificity = 0.58, sensitivity = 0.64. (**B**) ROC curve for Boruta based feature reduction method, AUC = 0.60, specificity = 0.47, sensitivity = 0.48. (**C**) ROC curve for CPH based feature reduction method, AUC = 0.55, specificity = 1.00, sensitivity = 0.18. (**D**) ROC curve for LASSO based feature selection method, AUC = 0.50, specificity = 0.26, sensitivity = 0.91. (**E**) ROC curve for the proposed risk-score based feature fusion method, AUC = 0.84, specificity = 0.68, sensitivity = 0.91.

Comparing the feature reduction methods using DeLong test, the performance of the proposed risk score-based method was significantly higher than PCA (0.84 vs. 0.60, *p*-value = 0.044, FDR adjusted *p*-value = 0.044), Boruta (0.84 vs. 0.60, *p*-value = 0.040, FDR adjusted *p*-value = 0.044), Cox-regression methods (0.84 vs. 0.55, *p*-value = 0.0086, FDR adjusted *p*-value = 0.017), and LASSO (0.84 vs. 0.50, *p*-value = 0.0062, FDR adjusted *p*-value = 0.017). The results suggest that a risk score model, which is based on probabilities calculated by multiple individual small models, gave the best performance compared to other models. The ROC curves for four traditional feature reduction methods (PCA, Boruta, CPH, and LASSO) and the proposed risk score-based model are shown in Fig. 5.

## Discussion

As deep transfer learning is becoming increasingly popular in medical imaging studies, there is an urgent need for identifying an optimal feature reduction and fusion method which can combine the information from traditional radiomics and transfer learning features. In this study, we proposed a risk score-based feature reduction and fusion method for a medical imaging-based model for PDAC prognosis. We discovered that the proposed risk score-based method had a significantly better prognosis performance than those of traditional supervised and unsupervised methods, increasing AUC by at least 40% (From 0.60 using PCA to 0.84). This result is consistent with previous studies, which have shown that ensemble methods can outperform traditional feature-wise selection models^50–52^.

As deep transfer learning increasingly plays a vital role in medical image analysis, the curse of dimensionality is becoming more acute in radiomics-based prognosis models^1^. Supervised feature reduction methods such as univariate CPH and Boruta have difficulties in balancing false positive rate and statistical power. By testing 1,463 features (1,428 PyRadiomics features and 35 LungTrans features) using univariate CPH, the probability of having at least one false positive (FWER) is higher than 99%. Hence, supervised feature reduction methods may lose their significance as feature banks continue to grow in size. In addition, PCA, an unsupervised method, wasn’t able to boost the prognosis performance due to the inherent noise in image features. Feature reduction using correlation cut-off with LASSO was previously used in a similar study for Glioblastoma prognosis^31^, but this method also failed in our independent test cohort in terms of performance. On the other hand, ensemble methods, which use multiple models to generate risk scores, may overcome these limitations of the traditional feature reduction methods^53,54^. Additionally, since risk scores were generated using a nonlinear classifier (Random Forest), they were in fact nonlinear mappings from the original feature space, providing better fits for patients’ survival patterns leading to higher AUC.

It is worth to note that although there were high Pearson correlation coefficients between certain transfer learning and PyRadiomics features, most deep radiomics features have weak linear relationships with PyRadiomics features. The nature of PyRadiomics features and LungTrans features is different. A PyRadiomics feature is extracted using a predefined formula from medical images while LungTrans features were extracted using parameters fine-tuned by lung CT images. This result suggests that the relationship between transfer learning and PyRadiomics features was more complementary than replacement. Thus, we hypothesized that fusing these two feature banks might provide more information to the prognosis model. Future studies can further test the associations between conventional radiomics features and transfer learning features from different pretrained models. A thorough understanding of these associations will provide a steady base for developing more sophisticated and advanced feature fusion methods, which may further improve the prognosis performance for different cancer types.

Although the proposed risk score-based method outperformed traditional approaches, it had limitations. First, compared to supervised methods where certain biomarkers can be identified during the process, the risk score method is hard to interpret since the stacked model is based on the results (probabilities) from other models. Although using intuitive algorithms such as logistic regression instead of Random Forests, one may derive the final prognosis probability (risk score) from original features using mathematical formulations, it would be a complicated task. Second, although lung cancer and pancreatic cancer are both adenocarcinomas, they are different in that pancreatic cancer tends to exhibit much more stromal reaction thus the features relevant to prognosis might be expected to be different. The effect of this on the transfer learning model is uncertain and further validation with a variety of adenocarcinoma types may be of interest to see if there are transfer learning features invariant across tumour types. Third, for practical applications, a model must include other known prognostic factors. In this case of pancreatic cancer, this includes variables such as age, tumour size, grade, and stage. Although it has been shown that none of these clinical variables is prognostic of overall survival in PDAC patients^8^, nor adding them to radiomic features improves the prognostic model^8^, further work is necessary to incorporate these into a practical prognostic model for PDAC. Forth, the aim of this paper was primarily to explore approaches to fuse radiomics and transfer learning features. We recognize that validation with a larger cohort with careful attention to covariates will be required for practical application and examining the effectiveness of the proposed feature fusion method.

## Conclusion

Deep radiomics features are complementary to conventional radiomics features. Through the proposed risk score-based prognosis model by fusing deep transfer learning and radiomics features, prognostication performance for resectable PDAC patients showed significant improvement compared to that of the traditional feature fusion and reduction methods.

## Data Availability

The datasets generated and/or analyzed during the current study are available from the corresponding author on reasonable request pending the approval of the institution(s) and trial/study investigators who contributed to the dataset.
